# Adaptations in the role of pharmacists under the conditions of the COVID-19 pandemic: a systematic review and meta-analysis

**DOI:** 10.1186/s12913-023-09071-w

**Published:** 2023-01-24

**Authors:** Dan Kambayashi, Toshie Manabe, Masayoshi Hirohara

**Affiliations:** 1 Laboratory of Pharmacy Practice, Center for Education and Research on Clinical Pharmacy, Showa Pharmaceutical University, Machida, Tokyo 194-8543 Japan; 2 Department of Medical Innovation, Nagoya City University Graduate School of Medicine, Nagoya, Aichi 467-8601 Japan; 3 Center for Clinical Research, Nagoya City University West Medical Center, Nagoya, Aichi 462-8508 Japan

**Keywords:** Pharmacist, Pharmacy practice, COVID-19, Education, Emerging infectious diseases, Systematic review

## Abstract

**Background:**

Community pharmacists actively engage in managing the health of local residents, but the COVID-19 pandemic has necessitated rapid adaptations in practice activities.

**Objectives:**

We sought to identify the specific adaptations in practice and the expanded roles of community pharmacists in response to the COVID-19 pandemic.

**Methods:**

We conducted a systematic review of published studies reporting the tasks of pharmacists in community pharmacies or who were involved in pharmacy practices addressing the pandemic. Two investigators independently searched PubMed (December 2019–January 2022) for eligible articles. We conducted a meta-analysis to measure the frequencies of practical activities by pharmacists in response to COVID-19.

**Results:**

We identified 30 eligible studies. Meta-analysis of these studies found that the most commonly reported adaptation in pharmacist practice activities was modifying hygiene behaviors, including regular cleaning and disinfection (81.89%), followed by maintaining social distance from staff and clients (76.37%). Educating clients on COVID-19 was reported by 22 studies (72.54%). Telemedicine and home delivery services were provided to clients by 49.03 and 41.98% of pharmacists, respectively.

**Conclusions:**

The roles of community pharmacists in public health activities have adapted and expanded in response to COVID-19, notably by incorporating public health education activities.

**Supplementary Information:**

The online version contains supplementary material available at 10.1186/s12913-023-09071-w.

## Background

As of 3 July 2022, the cumulative numbers of COVID-19 of cases and deaths worldwide have exceeded 546 million and 6.3 million, respectively [1]. Although the rates of COVID-19 infections and deaths have begun to decrease globally, the number of infections and deaths continues to grow in some countries and regions [1].

Pharmacists working in community pharmacies are known to support community health care as the first healthcare providers for community residents, especially during the COVID-19 pandemic [2]. In fact, our previous study indicated that many community pharmacists in Japan often provided the initial COVID-19-related consultations during the early phases of pandemic [3]. Several studies reported that in response to COVID-19, in addition to supplemental hygiene activities such as regular cleaning and disinfection of the pharmacy [3–13], community pharmacists were required to provide public health education to the community [14], and other various public health services such as home delivery services to clients [3–11] and remote explanation of medications [10, 12, 15–19].

Understanding the adaptations in pharmacists’ practice during the COVID-19 pandemic is crucial to strengthening the role of pharmacists as health partners to the community and for developing effective countermeasures to COVID-19 and potential future infectious disease pandemics. Previous reviews have mentioned the potential for community pharmacists to play an important role in the COVID-19 pandemic by taking on a variety of new roles that complement their existing work [20–22]. However, no comprehensive, systematic review and meta-analysis focusing on pharmacists’ practice during the era of the COVID-19 pandemic has been conducted.

The purpose of the present study is to examine the adaptations to the practice of community pharmacists in response to COVID-19 conditions, and how these adaptations contributed to public health and infection preventions, in an effort to provide an evidence base for discussing the role of pharmacists in future pandemics due to emerging infectious diseases.

## Methods

This systematic review and meta-analysis was conducted according to the Preferred Reporting Items for Systematic Reviews and Meta-Analyses (PRISMA) statement and the statement by the Meta-analysis of Observational Studies in Epidemiology (MOOSE) group [23, 24] (see Additional file 1).

### Eligibility criteria and outcome measures

Studies from the PubMed database fulfilling the following selection criteria were included in the meta-analysis: (1) randomized clinical trials, observational studies, letters and commentaries in the IMRD (introduction, methods, results, discussion) format written in the English language; (2) with a study population of pharmacists or others involved in pharmacy practices regarding COVID-19; (3) with primary outcomes of practical activities performed by pharmacists for COVID-19; (4) with outcome variables of pharmacy practices regarding COVID-19 in categories of drug and information delivery, client education, regular cleaning and disinfecting, and structural ingenuity; and (5) with any secondary outcome variable. The exclusion criteria were as follows: (1) studies of practical activities such as equipment and disinfection to protect individual pharmacists; (2) studies without reporting of outcome variables; and (3) studies with insufficient or incomplete data. We selected all reported outcome variables from the extracted papers and selected same reported variables in these reports as the outcome variables in the present study.

The main focus of this review was community pharmacists. However, it is necessary to understand what happened in hospitals to fully understand the situation relating to community pharmacists.

### Information sources and search strategy

Two investigators (D.K. and T.M.) independently searched for eligible studies published in PubMed, and the Cochrane Library from 1 December 2019 to 31 January 2022. We used the following key words: “novel coronavirus” OR “new coronavirus” OR “emerging coronavirus” OR “2019-nCoV” OR “COVID-19” OR “SARS-CoV-2” AND “pharmacist” OR “chemist” OR “apothecary” OR “pharmaceutist” OR “druggist”. We also reviewed the reference lists of eligible studies using Google Scholar and performed a manual search to ensure that all appropriate studies were included.

### Data extraction

Two investigators (D.K. and T.M.) independently searched for eligible studies. Articles obtained from the search were stored in Citation Manager (EndNote 20; Thomson Reuters, New York, NY, USA). After removing redundant articles, we examined the titles, abstracts, and full-text articles. Then, we extracted the data for country, methodology, study design, study participants, study site, sample size, study period, and main focus of each study. Outcome variables were extracted into pre-designed data collection forms. Data accuracy was verified by comparing the collection forms of each investigator, any discrepancies were determined through discussion [25].

### Level of evidence

The level of evidence was determined based on the Grading of Recommendations, Assessment, Development, and Evaluations (GRADE) framework, which classifies the level of evidence for each outcome based on the risk of bias, imprecision, inconsistency, indirectness, and publication bias [26]. The authors classified the evidence level for each eligible study according to the revised grading system for recommendation in the evidence base guideline (see Additional file 2) [27].

### Data analysis

Throughout the meta-analysis, we calculated the prevalence of each outcome variable with 95% confidence intervals (CIs) using a random-effects model (generic inverse variance method). To assess the prevalence of the outcome variables among pharmacy practices, the standard error was calculated using the Agresti–Coull method [28]. Heterogeneity among the original studies was evaluated using the I^2^ statistic [29]. Publication bias was examined using a funnel plot. For all analyses, significance levels were two-tailed, and *p* < 0.05 was considered significant. All statistical tests were performed using Review Manager (RevMan) Version 5.4.1 (Cochrane Collaboration, Copenhagen, Denmark) [30].

## Results

### Study selection and characteristics

**Fig. 1:**
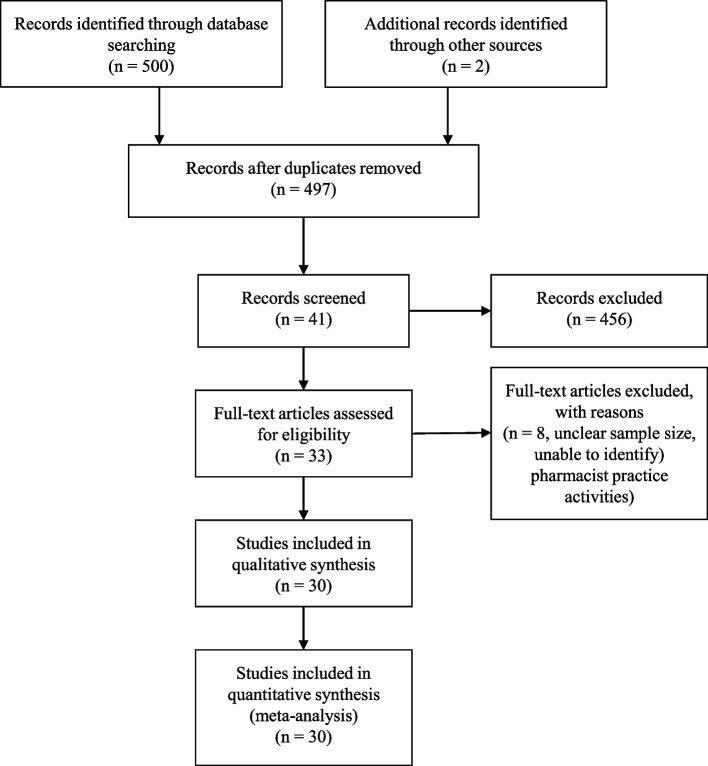
PRISMA flow diagram of selected articles. N is the number of articles

The initial database search identified 497 candidate publications. Of these, 30 studies [3–13, 15–19, 31–44] reported the outcome variables that met eligibility criteria (Fig. 1). Table 1 shows the characteristics of the included studies.

**Table 1:** Characteristics of eligible studies of pharmacy practices during the COVID-19 pandemic

Study, year	Country	Study design	Methodology	Study participants	Study site	Sample size	Study period	Main focus of the study	Level of evidence
Tortajada-Goitia B, et al., 2020 [15]	Spain	cross-sectional study	online survey	hospital pharmacists	hospital	185	March 14, 2020 – May 22, 2020	To analyze the status of the implementation and development of telepharmacy as applied to the pharmaceutical care of outpatients treated at hospital pharmacy services in Spain during the COVID-19 pandemic	2-
Hoti K, et al., 2020 [4]	Kosovo	cross-sectional study	online survey	community pharmacists	community pharmacy	264	April 1, 2020– April 30, 2020	Community pharmacists’ perceptions of COVID-19 related preventative measures	2-
Hussain I, et al., 2020 [31]	Pakistan	cross-sectional study	online survey	pharmacists	academia, retail and community pharmacy, hospital, and drug inspector	1149	March 30, 2020–May 22, 2020	To investigate pharmacists’ knowledge, attitudes, and practices regarding COVID-19 during the rapid rise period of the COVID-19 pandemic in Pakistan	2-
Abdel Jalil M, et al., 2020 [32]	Jordan	cross-sectional study	online survey	pharmacists	hospital or clinical, community pharmacy, academia and research	449	March 1, 2020–March 31, 2020	To assess the possible roles of Jordanian pharmacists in minimizing the stage of community transmission	2-
ElGeed H, et al., 2021 [7]	Qatar	cross-sectional study	online survey	pharmacists	community pharmacy	311	May 28, 2020 – June 18, 2020	To investigate the current practices, response preparedness and professional development needs of community pharmacists	2-
Meghana A, et al., 2021 [33]	India	cross-sectional study	online survey	pharmacists	academia, clinic and community pharmacy	24	Not provided	To gain rapid insights from pharmacy professionals in India regarding their roles and preparedness for the COVID-19 pandemic	2-
Bahlol M, et al., 2021 [5]	Egypt	cross-sectional study	in-person interview	pharmacists	community pharmacy	1018	April 8, 2020–April 19, 2020	To assess community pharmacies’ preparedness for the COVID-19 pandemic	2-
Wang R, et al., 2021 [34]	China	retrospective, observational study	electronic health records survey	COVID-19 patients admitted to ICU	hospital (ICU)	33	February 1, 2020–March 18, 2020	To share professional experiences on medication optimization and provide a feasible reference for the pharmaceutical care of critically ill patients with COVID-19	2-
Sum ZZ, et al., 2021 [6]	Australia	cross-sectional study	online survey	pharmacists	community pharmacy	137	April 1, 2020– April 30, 2020	To explore the current activities undertaken across various community pharmacy settings in relation to the safety of the workplace environment for staff and patients	2-
Zaidi STR, et al., 2021 [35]	UK	cross-sectional study	online survey	pharmacists	community pharmacy	206	May 4, 2020– May 30, 2020	To survey community pharmacists to understand their protective practices, professional and general wellbeing, and the delivery of pharmacy services during the COVID19 pandemic	2-
Muhammad K, et al., 2021 [36]	Pakistan	cross-sectional study	online survey	pharmacists	community pharmacy	393	April 10, 2020– April 30, 2020	To assess the knowledge, attitude, and practices of community pharmacists regarding COVID-19	2-
Baratta F, et al., 2021 [13]	Italy	observational study, cross-sectional study	serological test, self-report questionnaire	pharmacists	community pharmacy	286	July 1, 2020– July 31, 2020	To analyze the directives provided to pharmacists in 2020 regarding preventative safety measures to be adopted; to determine the number of pharmacists who came into contact with SARS-CoV-2 and relate this to the adopted preventative measures	2-
Yerram P, et al., 2021 [16]	USA	retrospective, observational study	electronic health records survey	patient involved with clinical pharmacy specialist	outpatient clinics	4022	March 1, 2020– May 31, 2020	To present an approach to restructuring clinical pharmacy services and providing direct patient care in outpatient clinics during the pandemic	2-
Jovičić-Bata J, et al., 2021 [37]	Serbia	cross-sectional study	online survey	pharmacists	community pharmacy	392	April 1, 2020– May 31, 2020	To present work environment changes related to COVID-19	2-
Akour A, et al., 2021 [17]	Jordan	cross-sectional study	online survey	patients with chronic disease who use community pharmacies	social media platforms	431	May 1, 2020–August 31, 2020	To evaluate the effect of the COVID-19 lockdown while exploring the role of community pharmacists	2-
Giua C, et al., 2021 [12]	Italy	cross-sectional study	online survey	pharmacists	community pharmacy	169	April 8, 2020– April 16, 2020	To describe procedures and critical logistical-organizational issues encountered by Italian community pharmacists and to collect the main requests reported by patients to pharmacists	2-
Elsayed AA, et al., 2021 [8]	Egypt	cross-sectional study	online survey	pharmacists	community pharmacy	413	August 1, 2020–August 30, 2020	To describe antibiotic misuse and its contributing factors; to measure pharmacists’ application of infection preventive practices during the pandemic	2-
Nguyen HTT, et al., 2021 [38]	Vietnam	cross-sectional study	mailing survey	community pharmacists	community pharmacy	1023	June 1, 2020– August 31, 2020	To survey the knowledge, attitudes, and practices of pharmacists regarding the COVID-19 pandemic	2-
Novak H, et al., 2021 [10]	Croatia and Serbia	cross-sectional study	online survey	community pharmacists	community pharmacy, social media platforms	574 (Croatia: 328, Serbia: 246)	June 1,2020–August 9,2020 (Croatia), July 1,2020–September 8, 2020 (Serbia)	To explore and compare the community pharmacists’ roles, practices, implemented safety measures, and psychological toll during the COVID-19 pandemic	2-
Wang D, et al., 2021 [19]	China	retrospective study	electronic health records survey	patients treated with pharmacotherapeutic intervention by a clinical pharmacist	hospital	349	February 5, 2020–March 10, 2020	To evaluate the usefulness of clinical prevention and control measures of clinical pharmacists	2-
Kambayashi D, et al., 2021 [3]	Japan	cross-sectional study	online survey	pharmacists	community pharmacy	1137	October 1, 2020– October 31, 2020	To conduct a nationwide survey among pharmacists to assess whether community pharmacists can contribute to reducing infections and the indirect secondary harm caused by COVID-19	2-
Yılmaz ZK, et al., 2021 [11]	Turkey	cross-sectional study	online survey	pharmacists	community pharmacy	393	May 1, 2020–July 31, 2020	To assess the knowledge, attitudes, and impressions of community pharmacists about COVID-19 and the factors affecting them	2-
Al-Daghastani T, et al., 2021 [39]	Jordan	cross-sectional study	online survey	community and hospital pharmacists	social media platforms	311	July 1, 2020– July 31, 2020	To analyze the role of pharmacists during the COVID-19 pandemic; to measure pharmacists’ attitude toward COVID-19 safety measures	2-
Kua KP, et al., 2021 [18]	Malaysia	cross-sectional study	online survey	pharmacists	community pharmacy	217	May 1, 2020– July 31, 2020	To examine community pharmacists’ views on their work environment, policies and preparedness for safe retail patronage to prevent the transmission of COVID-19	2-
Kassem AB, et al., 2021 [9]	Egypt	cross-sectional study	online survey	community pharmacists	community pharmacy	381	April 14, 2020– June 3, 2020	To evaluate the sources of knowledge and readiness of community pharmacists facing the COVID-19 early outbreak, to identify how far precautionary measures were applied in community pharmacies	2-
Elayeh E, et al., 2021 [40]	Jordan	cross-sectional study	online survey	residents	social media platforms	1179	March 26, 2021– April 16, 2021	To evaluate the type of drugs and treatments used to self-medicate/self-care during the pandemic, the reasons behind their self-medication, the sources of information and the factors affecting their practices	2-
Okuyan B, et al., 2021 [41]	Turkey	cross-sectional study	online survey	community pharmacists	community pharmacy	1098	May 21, 2020– May 29, 2020	To identify community pharmacist-led cognitive services and precautions taken related to COVID-19, perceived enablers and barriers related to pharmaceutical services and burnout level during the COVID-19 pandemic	2-
Mukattash TL, et al., 2022 [42]	Lebanon	cross-sectional study	online survey	home-treated COVID-19 patients	online questionnaire platform	279	October 2020	To explore the experiences and views of COVID-19 patients towards pharmaceutical care services provided during their infection	2-
Patel J, et al., 2022 [43]	USA	cross-sectional study	telephone survey	patients who received a pharmacist-provided COVID-19 test at a large-chain community pharmacy	contacted by telephone	622	May 1, 2020–June 14, 2020	To determine the local impact of community pharmacist-provided COVID-19 testing	2-
Alnajjar MS, et al., 2022 [44]	UAE	cross-sectional study	online survey	community and hospital pharmacists	hospital, community pharmacy	376	August 1, 2020–January 31, 2021	To evaluate pharmacists’ knowledge about and practice in the global COVID-19 pandemic	2-

Abbreviations: *ICU* Intensive Care Unit

Of the 30 studies, 27 were cross-sectional studies [3–13, 15, 17, 18, 31–33, 35–44] and 3 were retrospective observational studies based on electronic health records [16, 19, 34]. Although most survey participants in the cross-sectional studies were pharmacists, several studies included customers or patients using community pharmacies [17, 43], home treatment patients [42], and community residents [5] whose responses also indicated pharmacy practices regarding COVID-19. In this study, the largest number of studies on pharmacists’ practices regarding COVID-19 came from the West Asian region with 9 studies [7, 11, 17, 32, 39–42, 44], the most common country of origin for studies was Jordan [17, 32, 39, 40].

### Evaluation of pharmacy practice activities for COVID-19

Table 2 presents the major outcomes of studies of pharmacist practices regarding COVID-19.

**Table 2:** Main outcomes of pharmacists’ practices adapting to the COVID-19 pandemic

Study, year	Main outcome
Tortajada-Goitia B, et al., 2020 [15]	Implementation of pharmaceutical teleconsultations before delivery of medications (87.6%)
Hoti K, et al., 2020 [4]	Pharmacists were actively involved in counselling and educating patients in regard to COVID-19 treatments (91.7%) The pharmacy is cleaned and disinfected every day regularly (76.9%) Distance between pharmacy staff and patients is 2 m (67.0%) No more than one patient is allowed in the pharmacy at the same time (61.0%) Persons who are not pharmacy staff are not allowed to enter inside areas of the pharmacy (54.5%)
Hussain I, et al., 2020 [31]	Avoid unnecessary close contact and practice social distancing and keep at least 1-m distance from patients and other healthcare workers (95.7%)
Abdel Jalil M, et al., 2020 [32]	Educating citizens about the nature of the disease in general (93.8%) Distributing awareness brochures about COVID-19 disease (30.3%)
ElGeed H, et al., 2021 [7]	Disinfect the work area like counter, touch screens, telephone handset, keyboard, etc. (94.2%) Keep the recommended distance (> 1.5 m) between you and your customers/patients (93.6%) Receive questions from customers/patients about the symptoms of COVID-19 infection (84.2%)
Meghana A, et al., 2021 [33]	Patient communication materials prepared and distributed (41.7%)
Bahlol M, et al., 2021 [5]	Surfaces cleaned regularly (99.5%) Patient education on common COVID-19 symptoms (98.2%) Physical distance (95.5%) Prevention of customer crowding (94.7) Define a specific area for customers having suspected symptoms (64.0%) Home delivery service (49.1%)
Wang R, et al., 2021 [34]	Medication recommendations for patients with COVID-19 (66.7%)
Sum ZZ, et al., 2021 [6]	Frequently touched surfaces such as countertops were cleaned regularly (95.6%) Social distancing methods (83.9%) Limiting the number of patients (65.7%) Adequate consultation with social distancing (57.7%) Provided restricted sections of the pharmacy for COVID-19 suspected patients (35.0%)
Zaidi STR, et al., 2021 [35]	Limited customers are allowed in a given time to maintain social distancing rules (80.6%) Do’s and Don’ts and clear instructions for customers are placed at the entrance of pharmacy using a visible poster (74.8%) Physical barriers are in place to ensure only limited customers are allowed at any given time to enforce social distancing (52.4%)
Muhammad K, (2021) [36]	Educate and inform patients regarding COVID-19 disease (84.7%)
Baratta F, et al., 2021 [13]	Created separate entrance and exit for the clients (93.7%) Surfaces in the pharmacy are regularly sanitized (90.9%)
Yerram P, et al., 2021 [16]	Implemented via telemedicine to patient (93.7%)
Jovičić-Bata J, et al., 2021 [37]	Limiting the number of units per purchase for clients to overcome the shortages of safety equipment, dietary products and medicines (85.4%)
Akour A, et al., 2021 [17]	Community pharmacists you visited compliant with preventative measures including social distancing, wearing gloves and masks (80.7%) Home delivery services (55.0%) Community pharmacist provided medical advice about your over-the-counter medications (50.3%) Phone consultations (30.2%) Community pharmacy that you visited had an isolation room for patients with suspected COVID-19 (7.9%)
Giua C, et al., 2021 [12]	Continuous disinfection of working surfaces (89.9%) Telephone consultation implementation (59.2%) Home medicine delivery service through independent pharmacy organization (44.4%)
Elsayed AA, et al., 2021 [8]	Counseling inside pharmacy (96.4%) Regular sanitization for surfaces (89.8%) Home delivery (64.2%) Printed poster or flyers (33.7%)
Nguyen HTT, et al., 2021 [38]	Whether or not customers have asked questions involving COVID-19 (89.4%) Maintain at least one-meter distance between pharmacists and customers (85.2%) Deliver medicines to customers’ doors (42.0%)
Novak H, et al., 2021 [10]	Complete disinfection of working areas (82.1%) Frequent counseling activities related to public health issues such as preventive measures against COVID-19 infection (77.4%) Home delivery (26.7%) Online counseling (17.8%)
Wang D, et al., 2021 [19]	COVID-19 medication-related problems (23.5%)
Kambayashi D, et al., 2021 [3]	Ventilate the room regularly (95.5%) Disinfect indoor items and equipment (88.7%) Alert and educate patients about COVID-19 (67.5%) Online medication and drug delivery (67.5%) Limit the number of patients allowed into the pharmacy (19.0%)
Yılmaz ZK, et al., 2021 [11]	Made arrangements to keep a distance of at least 1–2 m between patients (89.3%) Gave training on personal protection precautions against COVID-19 (85.0%) After each customer we wipe and disinfect the pharmacy counter (56.7%)
Al-Daghastani T, et al., 2021 [39]	Pharmacist is committed to social distancing (86.5%) Corrected false information about the COVID-19 pandemic (76.2%)
Kua KP, et al., 2021 [18]	Restricted the number of customers allowed to enter the pharmacy (63.0%) Using the pharmacy social media site to provide health-related advice and updates on COVID-19 (41.5%) Medication delivery (39.3%) Drive-through medication services for customers (33.3%) Remote consultations (31.1%)
Kassem AB, et al., 2021 [9]	Patient consultation (55.9%) Daily sanitization (35.4%) Home delivery (20.0%) Social distancing (10.0%) Using printed materials to offer COVID-19 awareness (6.8%)
Elayeh E, et al., 2021 [40]	Source of information regarding COVID-19 (43.4%)
Okuyan B, et al., 2021 [41]	Responding questions related to COVID-19 (86.3%)
Mukattash TL, et al., 2022 [42]	Obtained medical advice about medication from the community pharmacist (48.8%) Supplied with a treatment management plan (29.7%)
Patel J, et al., 2022 [43]	After the conversation with the pharmacist, felt more knowledgeable about managing coronavirus signs and symptoms (90.0%)
Alnajjar MS, et al., 2022 [44]	Educated their patients about COVID-19 (25.7%) Provided information to the public about ways of reducing COVID-19 transmission as well as offered home delivery service (14.3%)

Various public health activities were commonly carried out, although the activities reported by pharmacists differed. These outcome variables were integrated into similar categories to estimate the percentage of pharmacist practice activities.

Thirty studies had data on any of the items that met the eligibility criteria [3–13, 15–19, 31–44]. Among the 30 studies with data on the primary outcome, we used meta-analysis to estimate the proportion of pharmacy operations related to COVID-19 in the categories of drugs and information delivery, client education, cleaning and disinfecting regularly, and structural ingenuity for each item.

**Fig. 2:**
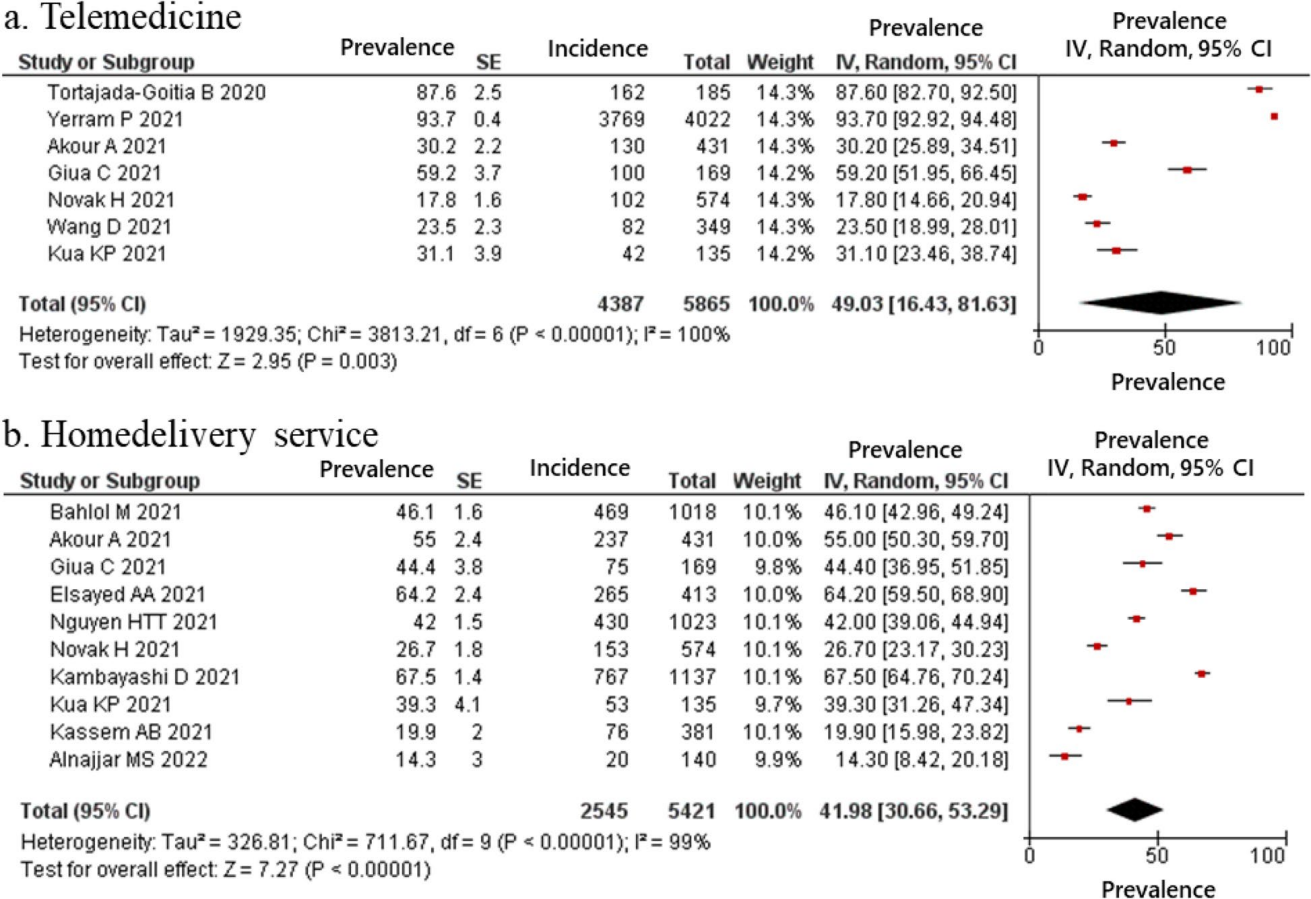
Proportion of pharmacy practices regarding COVID-19 in term of drugs and information delivery. (**a**) Telemedicine; (**b**) Homedelivery service

**Fig. 3:**
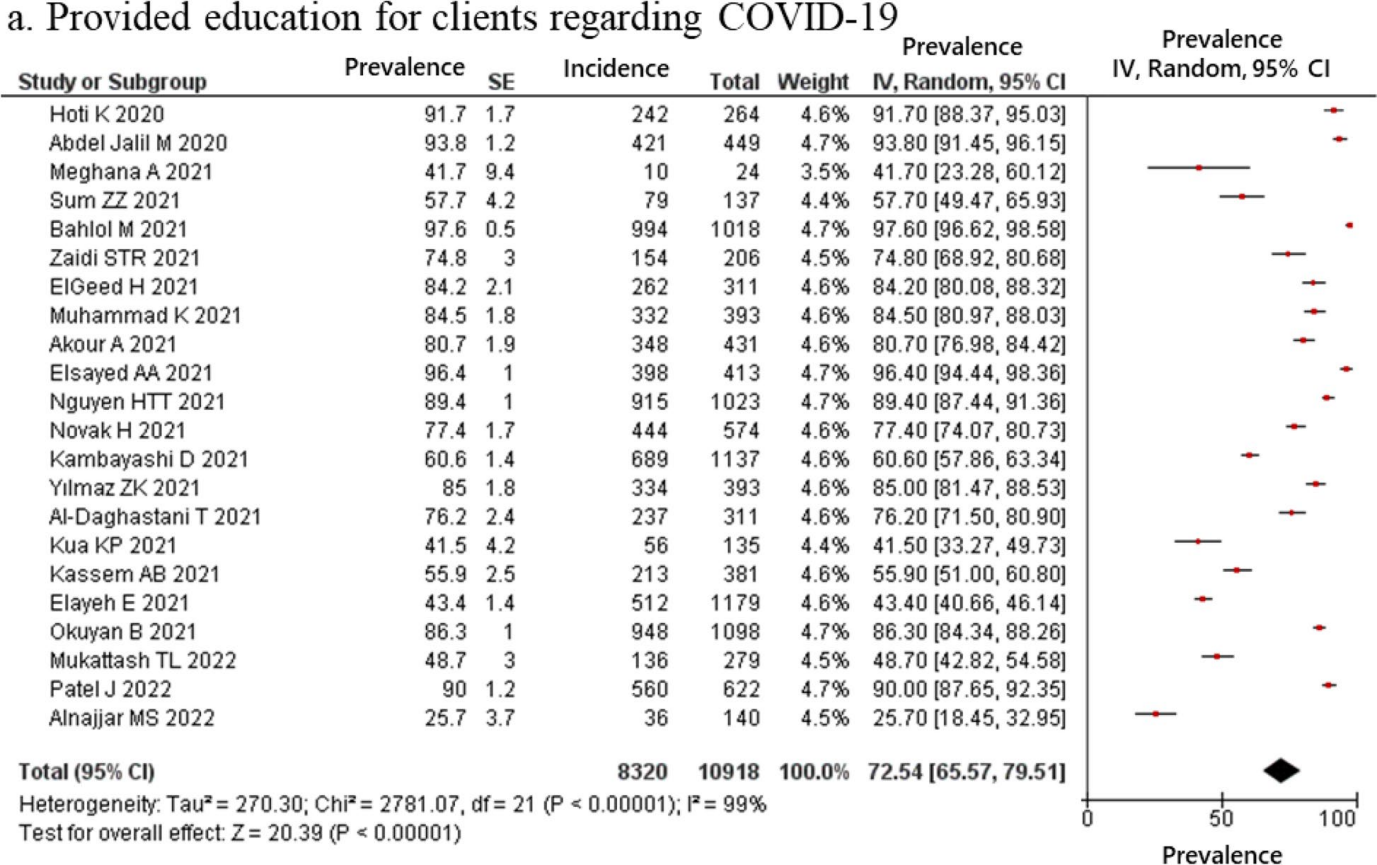
Proportion of pharmacy practices regarding COVID-19 in term of clients’ education. (**a**) Provided education for clients regarding COVID-19

**Fig. 4:**
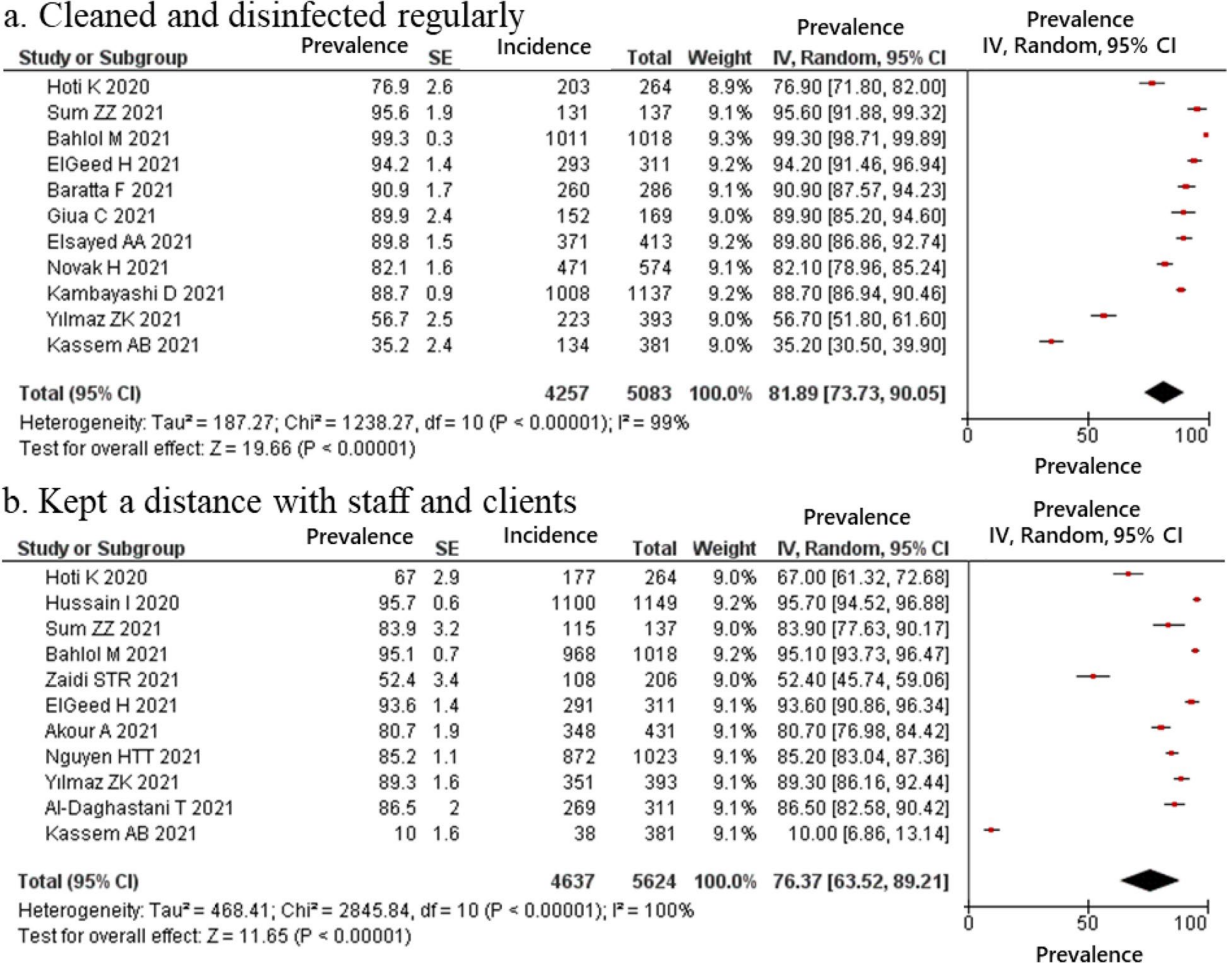
Proportion of pharmacy practices regarding COVID-19 in term of hygiene behaviors. (**a**) Cleaned and disinfected regularly; (**b**) Kept a distance with staff and clients

**Fig. 5:**
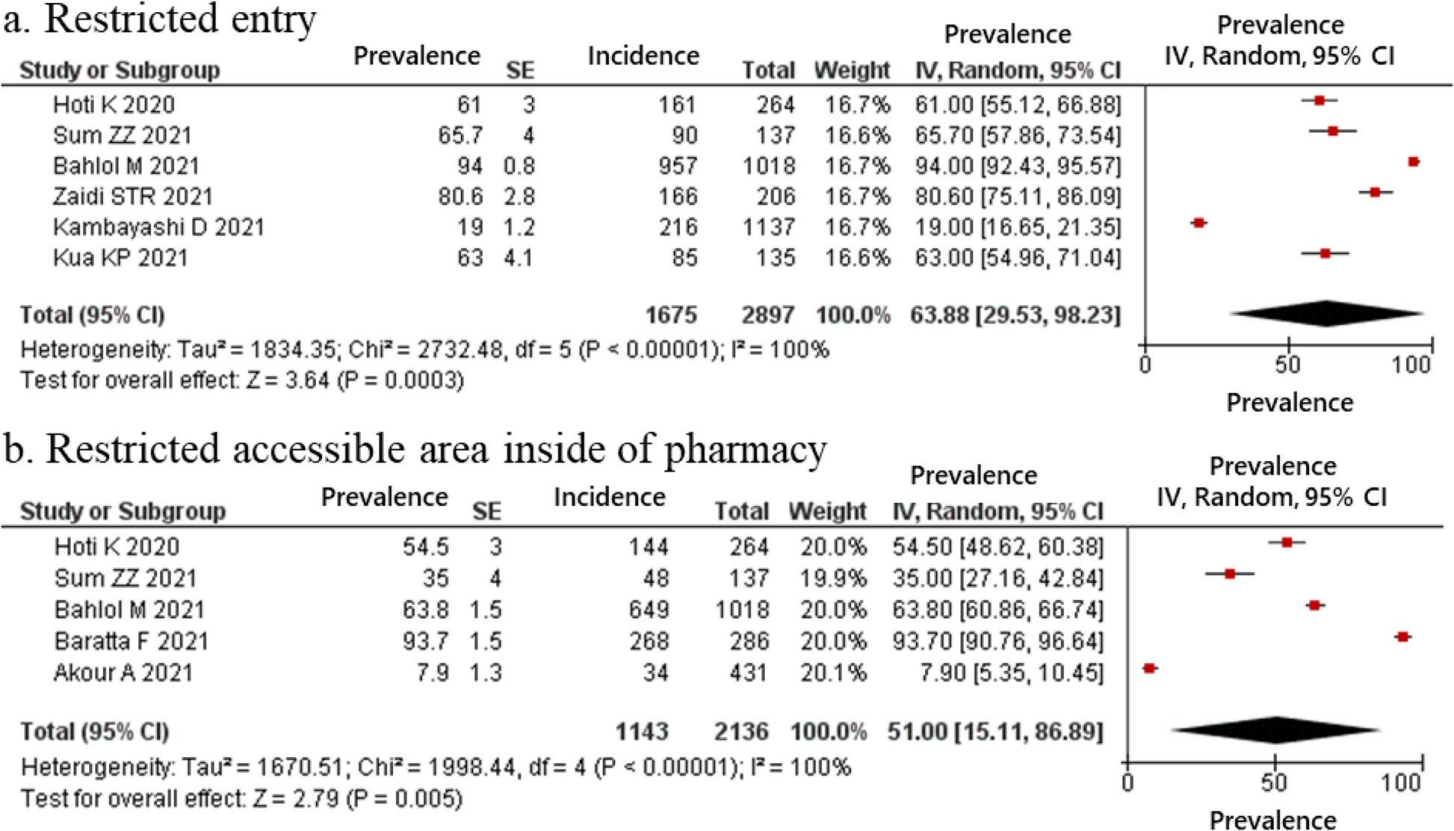
Proportion of pharmacy practices regarding COVID-19 in term of structural ingenuity. (**a**) Restricted entry; (**b**) Restricted accessible area inside of pharmacy

The estimated proportion of pharmacists who provided telemedicine and home delivery services, classified as drugs and information delivery, were 49.03 and 41.98% respectively (Fig. 2); those who reported providing education for clients regarding COVID-19 was 72.54% (Fig. 3). In the hygiene behaviors item, the estimated proportions of pharmacists who cleaned and disinfected regularly and kept social distance with staff and clients was 81.89 and 76.37%, respectively (Fig. 4). In the structural ingenuity item, 63.88 and 51.00% of the respondents implemented restricted entrance to the pharmacy and restricted access area in the pharmacy, respectively (Fig. 5).

### Secondary outcomes

Table 2 presents the various outcomes reported in each study. Of the 30 studies showing pharmacy practice for COVID-19, most focused on community pharmacists; four studies reported on only hospital and clinical pharmacists [15, 16, 19, 34].

Pharmacy practices other than the primary outcome included one report in which “clinical pharmacists recommended medication” for COVID-19 patients admitted to the ICU [34], as well as a report in which community pharmacists limited the number of items per client purchase to eliminate shortages of dietary supplements and medications [37].

## Discussion

The present study quantified the extent to which pharmacists are engaged in various public health activities for COVID-19 that augment health and infection prevention strategies for local residents. The most common practice activity of pharmacists in the response to COVID-19 was providing education for community residents.

COVID-19 is an emerging infectious disease with various factors, including prevention and treatment, that are evolving or were unknown, especially in the early days of the pandemic. People needed reliable information relating to COVID-19 and sought out healthcare professionals to provide them the appropriate and necessary information. In fact, in our previous study conducted in the first phases of the pandemic, community pharmacists received more questions from clients regarding COVID-19 than questions regarding drugs and medications [3]. Pharmacists are trusted community health care providers and are not only required to provide consultations as drug experts, but also to provide the necessary information to community residents in an easy-to-understand manner [45]. A scoping review of the role of pharmacists during the COVID-19 pandemic likewise reported that the pharmacist’s primary role was to provide information and counseling to patients [20]. Especially in an emergency situation in the setting of an emerging infectious disease pandemic, pharmacists must adjust their role to place greater emphasis on providing necessary information, conducting consultations and educating local residents. In addition, to augment the information publicly accessible via the media or the internet [3, 9, 36, 39], pharmacists have greater access to reliable scientific information from international organizations such as the World Health Organization and scientific and medical evidence than do the public they serve [4, 11, 32, 35, 41]. This education is the most effective and crucial way to provide information to local residents. In fact, the educational programs conducted by pharmacists for older adults can focus on both the drug regimens for management of their chronic diseases as well as simultaneously on precautions for the personal hygiene management and the necessity of securing physical distance to assist in the prevention of COVID-19 [5, 7, 8, 10, 12, 32, 41]. Some pharmacists’ practice activities adapted to include creating brochures and effectively using visual posters to educate clients about COVID-19 [8, 33, 35]. The results of the present study showing the high frequency of pharmacists providing education to local residents may result from their high level of understanding and motivation of this aspect as a major part of their professional role.

To impede the spread of the COVID-19 epidemic and to curb the impact of many unreliable or intentionally misleading news stories, community pharmacies in Italy worked with the Italian Ministry of Health and others to provide information to the local population on public health responses [13]. This “fake news” not only spreads inaccurate knowledge but has also been reported to have negative health effects on people, including various psychological disorders and fatigue [46]. In many countries, the role of pharmacists has shifted from targeting a product base to providing a variety of nonprescription services for patients [45], and new pharmacist services must continue to expand to reflect new social demands as well as historical changes. As trusted health care providers in the community, pharmacists must provide scientific messages in an easy-to-understand manner and contribute to the education and consultation of community residents regarding infectious diseases. A national survey in Japan reported that community residents consulted community pharmacies more about COVID-19 than medicines [3]. A cross-sectional study among Italian community pharmacy clients confirmed that overall satisfaction with pharmacies is high and that the role of community pharmacies is highly valued [47]. In many countries, community pharmacies are the first point of contact with the health care system for people with health-related problems or who simply need information or reliable evidence-based advice. This is essential even in a crisis such as the COVID-19 pandemic [48–50].

In the present study, the pharmacist practice reported at the highest frequency was regular cleaning and disinfection activities, followed by keeping social distance from staff and customers. Previous studies indicated that pharmacists, as medical professionals, have basic knowledge about COVID-19 infection control [31, 32, 38]. However, in the present study, the proportion of pharmacists providing home delivery service of medicines was low, even though pharmacist are well informed about the effectiveness of these services in preventing infection. Implementing a home delivery service necessitates additional requirements for the pharmacy, including human resources and distribution expenses. The low proportion of pharmacies offering this service in the pandemic may reflect these difficulties in implementation. Telemedicine is an effective means of offering consultations, especially during the conditions of infectious disease outbreaks. However, telemedicine may also be limited by institutional conditions as well as national infrastructure conditions. Among the studies that reported the adoption of telemedicine, high proportions of implementation were observed only by studies in the United States and Spain [15, 16], with few in low- and middle-income countries [10, 17–19].

The present study has some limitations, including those inherent to the nature of systematic reviews and meta-analyses. First, most of the papers included in this study were cross-sectional studies and did not necessarily have a high level of evidence. Because the reported outcomes varied among the studies, the number of studies focusing on each practice varied in this meta-analysis. Second, for the purposes of this analysis it was necessary to clearly organize the activities and focus the wide range of pharmacists’ community activities on the main action items. The categories of community practice activities for COVID-19 were therefore limited to only the items shown in the target criteria, we selected all reported outcome variables from the extracted papers and selected outcomes variables for the present study. Unreported pharmacy practices may exist in each study due to the survey’s focus on defined outcome variables. Third, the published articles included in the study were from only a few countries and did not broadly report practices in many countries. In addition, because the individual observational studies were conducted at different times of the year, we were unable to observe differences in pharmacy practice activities by infection status. Despite these restrictions, the present study is a meta-analysis of pharmacists’ practical activities related to COVID-19, showing the potential of pharmacists and providing important insights for expanding the role of pharmacists in the future.

Some countries have allowed pharmacists to expand their duties to include practices outside the scope of this study, such as COVID-19 vaccination and prescribing oral therapeutics [51–53]. However, this is not the case everywhere. We therefore focused on COVID-19-related activities that can be carried out regardless of national systems and backgrounds, and particularly provision of education and consultation by pharmacists. In addition to being involved in regular medication guidance, this work takes advantage of the convenience of pharmacies, where community residents can easily consult with pharmacists, providing rapid access to advice.

## Conclusions

In response to the conditions imposed by the COVID-19 pandemic, pharmacists adapted their practices to engage in various public health activities that have contributed to promoting health and preventing infection for their community residents. The education of local residents is the key element emphasizing the strength of their professional role in the response to COVID-19. Pharmacists have the potential to provide easy-to-understand scientific messages related to infectious diseases, and therefore contribute to protecting the health of local residents.

## Supplementary Information

**Additional file 1: ** PRISMA checklist.

**Additional file 2: ** Classification standard of the evidence level.

## Data Availability

All relevant data are included within the paper and the Supporting Information file.
