# Supplementary material for: Adaptations in the role of pharmacists under the conditions of the COVID-19 pandemic: a systematic review and meta-analysis
Source: BMC Health Serv Res. 2023 Jan 24;23:72. doi: 10.1186/s12913-023-09071-w (PMC9870779; doi:10.1186/s12913-023-09071-w)
Supplement: Supplementary file 2 — Additional file 2: Classification standard of the evidence level. [file 12913_2023_9071_MOESM2_ESM.docx]

**Additional file 2:** Classification standard of the evidence level.

| Level of Evidence | Study |
| --- | --- |
| 1++ | High quality of meta-analysis, systematic reviews of RCTs, or RCTs with a very low risk of bias |
| 1+ | Well controlled meta-analysis, systematic reviews, or low risk of bias |
| 1- | Meta-analysis, systematic reviews or RCTs, or RCTs with a high risk of bias |
| 2++ | High quality of systematic reviews of case-control or cohort studies  Or High quality of case-control studies with a low risk of confounding, bias or chance and a high probability that the relationship is causal |
| 2+ | Well controlled case-control or cohort studies with a low risk of confounding, bias or chance and a moderate probability that the relationship is causal |
| 2- | Case-control or cohort studies with a high risk of confounding, bias or chance and a moderate probability that the relationship is not causal |
| 3 | Non-analytic studies, eg case reports, case series |
| 4 | Expert opinion |

RCT, randomized controlled trial
